# Modeling of Tau-Mediated Synaptic and Neuronal Degeneration in Alzheimer's Disease

**DOI:** 10.4061/2010/573138

**Published:** 2010-08-24

**Authors:** Tomasz Jaworski, Sebastian Kügler, Fred Van Leuven

**Affiliations:** ^1^Experimental Genetics Group, Department of Human Genetics, KULeuven-Campus Gasthuisberg ON1-06.602, Herestraat 49, 3000 Leuven, Belgium; ^2^Center of Molecular Physiology of the Brain (CMPB), Department of Neurology, University Medicine Göttingen, Waldweg 33, 37073 Göttingen, Germany

## Abstract

Patients suffering from Alzheimer's disease (AD) are typified and diagnosed postmortem by the combined accumulations of extracellular amyloid plaques and of intracellular tauopathy, consisting of neuropil treads and neurofibrillary tangles in the somata. Both hallmarks are inseparable and remain diagnostic as described by Alois Alzheimer more than a century ago. Nevertheless, these pathological features are largely abandoned as being the actual pathogenic or neurotoxic factors. The previous, almost exclusive experimental attention on amyloid has shifted over the last 10 years in two directions. Firstly, from the “concrete” deposits of amyloid plaques to less well-defined soluble or pseudosoluble oligomers of the amyloid peptides, ranging from dimers to dodecamers and even larger aggregates. A second shift in research focus is from amyloid to tauopathy, and to their mutual relation. The role of Tau in the pathogenesis and disease progression is appreciated as leading to synaptic and neuronal loss, causing cognitive deficits and dementia. Both trends are incorporated in a modified amyloid cascade hypothesis, briefly discussed in this paper that is mainly concerned with the second aspect, that is, protein Tau and its associated fundamental questions.

## 1. Background

### 1.1. The Amyloid Problem

Amyloid peptides are derived from the amyloid precursor protein (APP) by sequential proteolytic cleavages by *β*- and *γ*-secretases [1, 2]. Amyloid peptides, particularly the longer A*β*42 species, aggregate into various and different chemical or physical entities, which are now considered the primary pathological agents in AD. Controversial experimental data leave nevertheless many questions open with regards to the exact composition and size of the amyloid oligomers in vivo [3–5]. Moreover, the precise molecular actions of the amyloid peptides and their aggregates remain unknown and enigmatic, largely because their molecular targets, or their specific receptors remain undefined. This issue raises more fundamental questions. For one, while the proteolytic enzymes and their mechanisms responsible for the generation of the amyloid peptides become well-known and understood at the molecular level, the eventual physiological function of the peptides is still questioned—and remains questionable. 

The original pathological importance ascribed to amyloid plaques was weakened, if not eliminated based on information gathered in transgenic models expressing mutant APP. We have advocated this hypothesis since we discovered early defects in cognition and LTP in APP.V717I mice [6–11]. Matters are, however, complicated further by recent data originating from several clinical trials that identified a large fraction of individuals in the control groups with considerable brain amyloid load using PET-imaging [12–14]. Obviously, this means that high-amyloid concentration in the brain is not per se incompatible with normal cognitive functioning in old age. This calls for other factors to be at least coresponsible for the cognitive demise in AD-forwarding protein Tau as the most obvious candidate. 

### 1.2. Tauopathy: Not Primary but Secondary in AD

Primary Tauopathy is recognized in a vast and still increasing number of CNS-disorders. The pathology is visualized postmortem as intraneuronal aggregates of protein Tau, known as neuropil treads and neurofibrillary tangles. These are similar if not identical to those observed in AD brain and mostly present without other associated pathological hallmarks, except inflammation markers. The large clinical variability among the primary Tauopathies stems from the presence of the Tau pathology in different types of neurons and in different brain-regions [15–18]. 

Biochemically, all Tauopathies consist of smaller and larger aggregates and fibrils that become intertwined to form larger tangles in the soma as well as in axons and dendrites of affected neurons. The aggregates consist almost exclusively of protein Tau, albeit as different isoforms in different diseases, that is, either Tau.3R or Tau.4R or as a variable admixture. Their widely different grading of phosphorylation is usually referred to as hyperphosphorylated Tau, denoted here as hP-Tau. Importantly, no accurate definition based on either the level or the specific phosphorylated residues can typify any Tauopathy exactly, because of the large variability with each primary disease. 

Most primary Tauopathies are sporadic. Rare familial cases of frontotemporal dementia (FTD) are linked to exonic and intronic mutations in the MAPT gene coding for protein Tau (chromosome 17). Although rare, their identification marked a major breakthrough and boosted interest in Tauopathy, because of the evident implication that Tauopathy itself is sufficient to cause cognitive decline and dementia [15, 16, 18–21]. 

Both intronic and exonic mutations have important mechanistical implications: (i) most expressed mutations are in the microtubule binding domain of Tau; (ii) intronic mutations promote or hinder splicing of exon 10 coding for the second repeat domain in the microtubule binding domain, skewing expression of isoforms to either Tau3R or Tau4R, but both with normal sequences and consequently normal binding-affinity for microtubules. 

The pathology resulting from intronic mutations in the MAPT gene can only be explained by effects on RNA splicing, eventually resulting in changes in absolute or relative levels of Tau isoforms that otherwise have normal wild-type protein sequences. The overexpression or overrepresentation of one or the other Tau isoform can then be extrapolated to the primary Tauopathies that are sporadic, as well as to sporadic AD as the most prominent secondary Tauopathy involving normal, wild-type Tau-isoforms. 

### 1.3. The Obligate Amyloid-Tau Relation

The major unresolved problem in AD is the mechanistic relation of amyloid and Tau pathology. Amyloid oligomers are proposed to precede, and eventually trigger the intracellular Tauopathy, likely by increasing the phosphorylation of protein Tau [11, 15, 17, 22]. While this hypothesis remains at this moment in time impossible to prove in sporadic AD cases, it is supported by extrapolation of observations in familial AD cases. Indeed, even in the most early onset familial AD that are caused by mutant Presenilin or mutant APP, and therefore by definition are “amyloid-triggered”, Tauopathy is always evident and codiagnostic. Experimentally, double and triple transgenic mouse models with combined amyloid and Tau-pathology prove the same intimate relation [23–26]. 

The genetic data imply that deranged Tau-microtubule interactions, caused either directly by the mutation, by phosphorylation, by increased absolute or by disturbed relative concentrations of Tau, all can contribute or even be sufficient to cause neurodegeneration in primary Tauopathies, that is, in the absence of amyloid pathology. Importantly, experimental decrease of the level of Tau in amyloid transgenic mice can actually ameliorate several clinical symptoms and defects [27]. The combined data further corroborate the hypothesis that protein Tau is essentially contributing to amyloid-induced cognitive defects. 

Not resolved are the identity and exact nature of the receptors and associated signaling pathways that lead from amyloid to Tau. These pathways obligatory involve kinases such as GSK3 [25, 26] that contribute, directly or indirectly to the phosphorylation of Tau at residues that affect its binding to microtubuli. This is thought, but not proven, to instigate its eventual aggregation into fibrils that are deposited locally as neuropil treads or transported to the soma and form the neurofibrillary tangles, and both types of aggregates occur in the same neurons. Nevertheless, Tauopathy does not per se cause immediate or even delayed neuronal death, as tangled and Tau-loaded neurons are observed in humans as well as in experimental models, that is, transgenic mice, zebra-fish, and flies [26, 28, 29]. 

The pertinent question to be answered then is: what chemical or physical form of protein Tau causes synapto- and neurodegeneration? 

## 2. Transgenic and Adeno-Associated Virus Models

Invaluable insight in the problems that we are concerned with, have resulted from analyzing different transgenic mouse models. Our research-group generated, characterized, and validated many different transgenic mouse strains and their bigenic combinations as preclinical models over the last two decades. So far none of the models recapitulates robustly the evident neurodegeneration observed in the brain of AD patients. 

We explored and reported on additional models to increase our understanding of this aspect of the pathogenesis of AD [11, 30]. Here, we review data and insights obtained with adeno-associated viral vectors that were engineered to express wild-type or mutant APP or protein Tau in pyramidal neurons of the hippocampus of wild-type mice. Thereby, we aimed to recapitulate aspects of AD pathology and related features that are not attainable in transgenic mice, particularly neurodegeneration.

**Figure 1:**
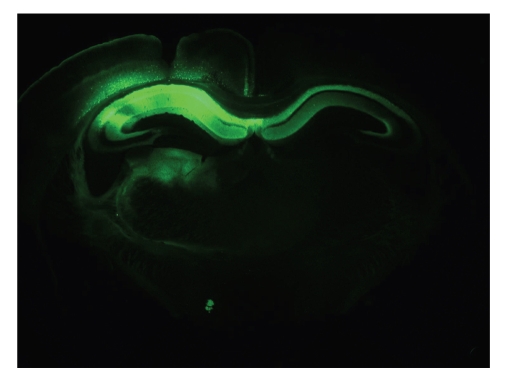
*Expression of EGFP following hippocampal injection of AAV1/2.EGFP is long-lasting.* Six months following intracerebral injection of the AAV.EGFP vector, the transgene remained expressed in the CA1/2 subfields of the hippocampus and in the deeper layers of the cortex. Expression of EGFP is also evident in and likely propagates along axonal projections of the fornix.

Among the different viral vectors available, we selected AAV because these have been demonstrated as excellent and safe tools for gene delivery into the CNS. Recent technical advances have improved AAV-based vectors as tools in neuronal research [31, 32]. The expression of the human transgenes used in our studies was controlled by the human Synapsin1 gene promoter, with constructs packed in chimeric capsids AAV1/2, expressing efficiently in pyramidal neurons of the CA1/2 subfield of the hippocampus (Figure 1). Control AAV vectors expressed EGFP in the designated neurons and areas that persisted for months without negative signs or symptoms (Figure 1) and [31, 32]. 

### 2.1. Amyloid Pathology without Neurodegeneration

We first constructed AAV vectors to recapitulate the amyloid pathology in the limbic region of wild-type mice. The constructs contained APP695 as the most abundant neuronal isoform, either as wild type sequence or as the engineered triple mutant (denoted APP.SLA). The incorporated Swedish, London and Austrian mutations are each associated with familial forms of AD [33–35]. 

Following intracerebral injection into wild-type mice, the APP.SLA mutant produced a gradual accumulation of APP and amyloid peptides in pyramidal neurons in the hippocampal CA1/2-region, as well as in the deeper layers of the neighboring cortex. Similar expression of wild-type human APP695 failed to produce any deposition of amyloid or of the related effects observed with mutant APP.SLA [30]. 

**Figure 2:**
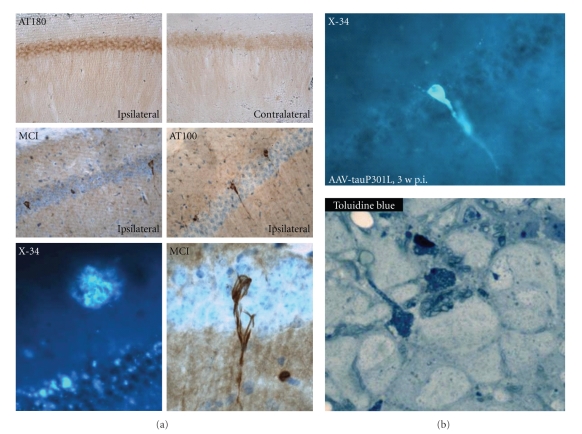
*Protein Tau is activated by and therefore downstream of amyloid pathology.* (a) Tau.P301L transgenic mice [36] were injected intracerebrally at age 1.5 months with either AAV-APP.SLA vector (10e8 tu) or with AAV-EGFP as control. At 6 months p.i. the expression of APP.SLA, unlike EGFP, led to formation of amyloid aggregates and plaques, as shown by histochemical staining with X34 (lower left panel). The mutant APP.SLA also strongly increased the phosphorylation of transgenic Tau.P301L resulting in the formation of neurofibrillary tangles already at this age of the Tau.P301L mice in pyramidal neurons of CA1: immunohistochemistry with Mab AT180 specific for phospho-T231-Tau (upper panels), Mab MC1 (conformational Tau-epitope) (middle panel left) and Mab AT100 (phospho-S212/T214) (middle panel right). Remarkably, the amyloid plaques and Tau pathological aggregates coexist in AAV-APP.SLA-injected Tau.P301L mice without causing appreciable neurodegeneration. (b) Intracerebral injection of AAV-Tau.P301L (10e8 tu) in wild-type mice produced extensive neurodegeneration of CA1 pyramidal neurons as shown before [11]. Aggregates of Tau or tangles were very rare although they can form in these neurons in the experimental conditions, as shown histochemically with compound X34 as sensitive probe for *β*-sheeted aggregated protein [12, 14]. Clearly, the paucity of tangles cannot explain the extensive neuronal cell death. Degenerating neurons share morphological features of necrosis and autophagy, with extensive vacuolization as demonstrated histochemically by toluidin blue staining (bottom panel).

Initially, from 3 weeks to 3 months postinjection (p.i.), amyloid immunoreactivity appeared as intracellular inclusions or vacuolar bodies inside pyramidal neurons. At 6 months p.i., amyloid plaques developed as immunoreactive and thioflavinS positive deposits in the hippocampus and cortex (Figure 2). At this late time-point, we also observed increased phosphorylation of endogenous mouse protein Tau, particularly at residues T181 and T231, both known to be substrate for GSK3. These phosphoepitopes, defined by antibodies AT270 and AT180 were differentially expressed, that is, in hippocampal pyramidal neurons (T231) and in dystrophic neurites around amyloid plaques (T181). 

In contrast, Intracerebral injection of AAV-APP.SLA not in wild-type but in Tau.P301L mice led to the formation of neurofibrillary tangles (NFT), demonstrating that the viral assault did not prevent protein Tau from forming tangles (Figure 2). Moreover, the combination demonstrated that besides the primary hit, also the receiving genetic background is of utmost importance. Most interesting for the interpretation of the data obtained with protein Tau, discussed in the next section, was the observation that at 6 months p.i. the phosphorylation of endogenous mouse Tau was increased and coincided with a minor but significant reduction in the number of neurons in the CA1 region of AAV-APP.SLA injected mice [30]. 

### 2.2. Tau-Mediated Pyramidal Neurodegeneration without Tauopathy

In second instance, we similarly expressed either wild-type Tau.4R or mutant Tau.P301L by AAV-vectors in wild-type mouse brain. In sharp contrast to APP-SLA, AAV-mediated expression of protein Tau resulted already at 3 weeks p.i. in marked neurodegeneration of pyramidal neurons in CA and adjacent cortical layers [30]. These findings were rather unexpected and we analyzed them in different directions, yielding novel and important insight into the mechanisms causing neuronal death by protein Tau. 

Most remarkably, we could not detect any major form of aggregates of protein Tau in pyramidal neurons that expressed either wild-type or mutant protein Tau. Extensive analysis by histochemistry and immunohistochemistry with a panel of indicator compounds and monoclonal antibodies to protein Tau, did not reveal any appreciable signs of formation of intraneuronal Tau-aggregates, either before or during or after the pyramidal neurons in CA1/2 succumbed [30]. 

The observed neurodegeneration was not due to massive overexpression of human protein Tau. The actual levels in hippocampal extracts were near-physiological and only about twice those of endogenous mouse Tau4R. Note that also in the AAV-APP.SLA model described in the previous section, the hippocampal levels of human APP.SLA were only about twice those of endogenous murine APP. In addition, expression of EGFP by the same AAV-type vectors proved harmless to pyramidal neurons over long time-periods (Figure 1) and [30, 31]. 

Intracerebral injection of 3- and 10-fold less AAV-Tau, with consequently less expression of human protein Tau, produced a graded, lesser loss of hippocampal neurons [30]. The AAV-Tau-induced neurodegeneration is thereby further demonstrated to be directly caused by and proportional to the near-physiological levels of protein Tau.

Consequently, the observed pyramidal cell-death is qualified as specific for human protein Tau. This conclusion holds up for wild-type Tau.4R and for mutant Tau.P301L, making the model at least conform the observations in familial FTD. Indeed, as discussed above, intronic and exonic mutations yield very similar clinical outcome, despite the expression of mutant or wild-type Tau4R, respectively. The actual contribution of the mutation to the mechanism underlying the pathogenesis requires a rational explanation in human FTD patients, as well as in the AAV-models. 

## 3. Mechanisms Underlying Tau-Mediated Pyramidal Neurodegeneration

Extensive analysis to define the mechanisms that underlie the observed Tau-mediated pyramidal neurodegeneration are described, summarizing reported data and preliminary data of work in progress. 

Similar AAV-mediated expression of a truncated version of protein Tau.4R, lacking the microtubule binding C-terminal domain, proved completely harmless and did not cause any neurodegeneration over similar time-periods [30]. The inherent conclusion must be that microtubule binding of protein Tau is essentially involved in the neurotoxic degenerative mechanism. Consequently, we must orient further analysis to mechanisms that involve axonal and dendritic transport over microtubuli, which is of paramount importance for all cells but especially for neurons with their intricate branching. 

Of note, microgliosis was observed to be intense, and spatially and temporally closely associated with AAV-Tau induced degenerating neurons [30]. This was most recently also reported in a similar model based on AAV vectors but in rats and for a different pathology in a different brain-region [38]. Previously, we have observed a similar relation of microgliosis to neurodegeneration in an unrelated model for hippocampal sclerosis, caused by conditional expression of p25, the truncated activator of cdk5 [39]. Also, in that model, intense neurodegeneration was not marked by aggregation of protein Tau, further strengthening our conclusion that cdk5 is not a major Tau-kinase in vivo [39–41]. 

The data originating from the AAV-models led us to conclude that not large aggregates of protein Tau cause neurodegeneration [30]. This confirms our observations in transgenic mice that show aggregation of Tau leading to Tauopathy in somata and neuropil, but not accompanied by marked neurodegeneration [25, 26, 36]. 

We proposed that neurons affected by Tauopathy can either engage in aggregation of Tau and thereby try to decrease the toxic species and hope to survive, or to enter the “path to death” by failing to aggregate protein Tau [11]. Clearly, the viral models comply with the transgenic models that aggregation of Tau and neurodegeneration are not closely linked. Thereby, questions are raised that are addressed in more detail in the next sections. 

### 3.1. What is the Neurotoxic Tau-Species? 

The classic concept that Tau-fibrils or tangles are neurotoxic in Tauopathies can be abandoned. We advocate that neurofibrillary tangles, like amyloid plaques are the final pathological hallmarks, but not the neurotoxic agents. The outcome fits independent observations that the extent of neuronal loss exceeds the number of NFT in patients with AD [42, 43]. In inducible Tg4510 mice, memory decline and neuronal loss are dissociated from tangle formation in time and brain region [44, 45]. 

In the AAV-Tau injected mice, phosphorylation of Tau was evident at many pathological epitopes, including those defined by antibodies AT8, AT100, AT180, and AT270 [30]. Despite the increased phosphorylation no aggregates of protein Tau were deposited in degenerating pyramidal neurons, analyzed histochemically or immunohistochemically. Biochemical analysis indicated the formation of low molecular weight aggregates [30] which need and deserve further analysis. 

Intriguingly, experiments whereby AAV-APP.SLA was injected intracerebrally not in wild-type mice but in transgenic Tau.P301L mice, produced not only amyloid plaques but also intracellular Tau aggregates. Nevertheless, no substantial neuronal loss was evident, substantiating our previous data of absence of marked neurodegeneration in Tau.P301L mice and Tau.P301LxGSK3b bigenic mice (biGT), despite extensive or even dramatic Tauopathy [26, 36]. 

The combined results from our experimental models and from a Drosophila model of Tauopathy [46] consolidates the thesis that neurotoxicity is not exerted by large aggregates of protein Tau but rather by Tau-species that are intermediate between normally phosphorylated protein Tau and the hyper-phosphorylated fibrils. The identity of the toxic Tau species, which we have termed “Tau-P*” [11, 30] remains to be defined—a challenge equaling that of the identification of the amyloid receptor. 

It must be remembered that Tau is subject to other posttranslational modifications, besides phosphorylation. Indeed, Tau can be ubiquitinated, truncated, glycosylated, glycated, oxidized, and, moreover, undergoes isomerization at proline residues. At any given moment protein Tau is present in various molecular forms, which most likely differ subtly in properties of binding to microtubuli, interaction with other proteins, binding to membranes, being transported for normal duty or for degradation. 

The decision whether neurons will enter the cell death path or protect itself by forming Tauopathy, must depend on the actual levels of protein Tau and on its modifications. The rapid accumulation of Tau-P* will lead to cell death, while more gradual accumulation of Tau-P* would result in formation of aggregates. Both pathways will be affected by various external factors, for example, inflammation, amyloid, stress, and so forth. In this concept, the aggregation of Tau acts as the “escape from cell death pathway”. Evidence from patients, whereby CA1 hippocampal neurons survive for decades despite neurofibrillary tangles [47] and experimental animals show tangle bearing neurons survive [26], even with lost membrane integrity [48]. 

### 3.2. Protein Aggregation, Impaired Clearance? 

Two major pathways are responsible for removal of damaged, unfolded, or aggregated cellular proteins: the ubiquitin/proteasome system and the autophagy/lysosomal system that also can remove aged or damaged organelles. Malfunction of autophagy can result in accumulation of protein aggregates that may contribute to the disease pathology in AD and other neurodegenerative disorders [49–52]. Stimulating autophagy pharmacologically, for example, by inhibition of mTOR, ameliorates cognitive deficits and reduces amyloid and Tau pathology in the 3xTg mouse model for AD [53]. Autophagosomes form in a stepwise process that requires transport over the microtubular network, which is known to be affected by protein Tau, a major microtubule-associated protein. 

In our mouse models, we did however not detect clear indications for problems with autophagy. Degenerating neurons in the AAV-Tau.P301L injected mice displayed as dark neurons with extensive vacuolization that could be taken to indicate a defective autophagic process. A similar facet of neurons we observed in an unrelated mouse model, that is, the p25 inducible mice that recapitulate hippocampal sclerosis [39]. The combined data do lend more support to the hypothesis that vacuolization is a correlate or consequence of the degenerative process, as an intrinsic mechanism needed to clean up defective organelles, protein aggregates or even entire dead or dying neurons. Consistent with this view is our observation in the AAV-Tau.P301L mice that vacuoles do not form early in the disease process but only after the neurons are already degenerating [11, 30]. Finally, we searched but did not find biochemical evidence for the conversion of LC3-I to LC3-II, which is generally accepted as the necessary step in and read-out of the increased formation of autophagosomes. 

### 3.3. Cell Cycle Reentry, or Not? 

After differentiation from neuronal precursors, neurons enter their post-mitotic state, while attempts to reenter the cell cycle is considered pathological and eventually result in cell death. In AD and other Tauopathies, cell-cycle related events have been suggested to be directly related to neurodegeneration. 

We tested this hypothesis in the AAV-Tau model by examining cell cycle-related markers. Various markers were increased, that is, CyclinD2, phosphorylated Retinoblastoma protein, proliferating cell nuclear antigen (PCNA), cyclin B1 [30]. However, intracerebral injection of AAV-Tau in cyclinD2 deficient mice showed no extra effect on neurodegeneration, suggesting that cell cycle reentry is not a pre-requisite for neuronal death [30]. 

### 3.4. Microtubular Transport

The only physiological function for protein Tau is binding to and stabilization of microtubules. Microtubules ensure cell shape and constitute roads of transport, a feature prominent in neurons with the most complex architecture of all cells. Microtubule dependent transport is ensured by families of motor proteins dyneins and kinesins, respectively for retrograde transport from distal processes towards soma and as plus-end directed motor for anterograde transport. 

The effect of protein Tau on transport appears to be dual. First, mutant Tau and hP-Tau can cause it to detach from the microtubules and decrease its ability to control microtubule dynamics. On the other hand, increased levels of protein Tau can saturate microtubules and hinder the “foot-stepping” of the motor proteins needed for axonal and dendritic transport. Both aspects of Tau-related transport deficits have been observed and both can fit into a model leading to “starving synapses” that eventually culminates in neuronal death. In this hypothesis, Tau-mediated neurodegeneration is likely to start in distal neuronal processes, that is, axons and apical dendrites, which degenerate progressively towards the cell soma [54]. 

Synaptic pathology could be an early defect in neurodegenerative Tauopathies [55] as proper functioning of distal synapses is totally dependent on adequate transport of essential cargos and uninterrupted provision of energy. Impaired oxidative phosphorylation and mitochondrial dysfunction has been observed in mouse models expressing mutant Tau, which was potentiated by mutant APP and Presenilin1 [56, 57]. 

In Tau.4R transgenic mice, we previously documented axonopathy and motor deficits that were rescued by coexpression of constitutively active GSK3b [37, 58]. The data explain how excess Tau in first instance blocks transport by covering microtubuli, while GSK3 acting as major Tau-kinase I phosphorylates Tau to detach it from the microtubules and relieves the transport blockade [37]. Interestingly, treatment of Tau.4R mice with lithium salts significantly increased the axonopathy, an effect that can be ascribed to its inhibition of GSK3b [59]. 

**Figure 3:**
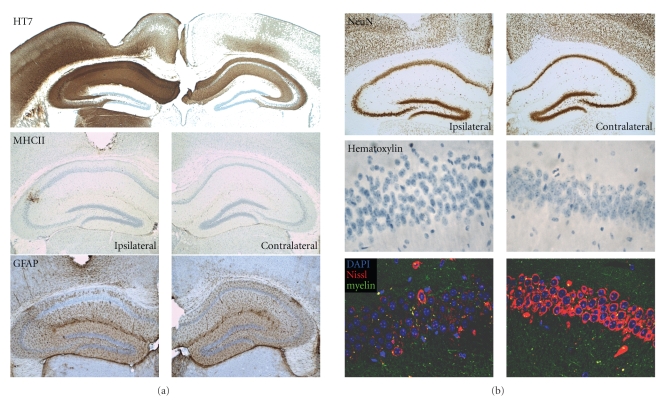
*Tau-mediated toxicity depends on the microtubule binding domain.* Intracerebral injection of AAV-Tau.255 (10e8 tu) into wild-type mice to express a truncated form of protein Tau devoid of the microtubule binding and C-terminal domains, did not cause appreciable neurodegeneration nor inflammation. Shown are immunohistochemical stainings at 3 months p.i. for human Tau (HT7), GFAP, and MHCII as indicators of astrogliosis and microgliosis, respectively [30]. Neurons expressing truncated protein Tau still maintain neuronal nuclei (NeuN) despite changes in nuclear morphology (hematoxylin) and in content of Nissl substance (lower panels).

**Figure 4:**
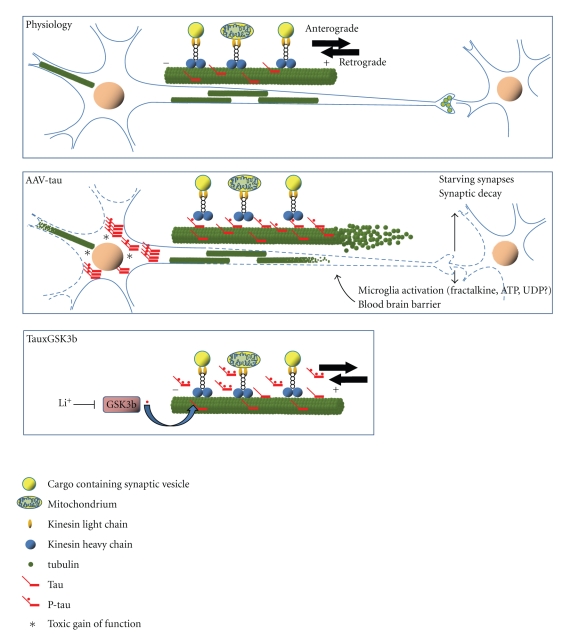
*Model for Tau-mediated neurodegeneration.* In normal conditions, protein Tau is regulated tightly at different levels: isoform expression, phosphorylation, microtubule binding, turnover, all needed to ensure normal transport along microtubules in axons and dendrites (upper panel). Increased expression of protein Tau, either genetically or pathologically in humans or experimentally in animal models will increase the amount of protein Tau bound to microtubules, thereby competing for and blocking the binding-sites needed for the motor proteins that carry out transport (middle panel). The resulting impairment in transport of any cargo, from synaptic vesicles and mitochondria to proteins, will impair any transport and energy-dependent processes at the synapses, which will extend and evolve into degenerating neuronal processes, and eventually lead to neuronal death. Defective synaptic transmission is then expected to be an early indication or symptom. Initially, small and loose aggregates or oligomers of protein Tau collect onto the microtubules, causing them to disintegrate or collapse. We also propose that the injured neuronal processes release proteins and factors, purposely or accidental that contribute to the activation of microglia and astroglia. The activated inflammatory cells secrete then factors that affect not only neurons but also other cells that constitute the unit blood-brain-barrier, provoking higher permeability, which further negatively affects neurons [11, 30]. Interesting is the connection to increased activity of GSK3, which rescued the axonopathy of Tau4R mice and the premature death of Tau.P301L mice by phosphorylating protein Tau and thereby detach it from the microtubuli to restore normal transport by motor proteins (lower panel) [26, 37]. Likewise, neither cell-death nor inflammation is provoked by AAV-Tau.255, devoid of the microtubule binding domain. The transgenic and viral models thereby underscore the microtubule binding of protein Tau as the common mechanism of action whereby protein Tau is causing neuronal demise and eventually neurodegeneration.

As discussed above, AAV-mediated expression of truncated Tau, lacking the microtubule binding domain did not cause neurodegeneration, in marked contrast to full length protein Tau (Figures 3 and 4) and [30]. The combined data from various approaches allow us to conclude that Tau mediated pathology is, at least in part, mediated by its microtubule binding properties. 

### 3.5. Inflammation

Chronic inflammation is evident in brain of AD patients and proposed to contribute essentially to the “vicious circle” leading eventually to neuronal death, brain atrophy and severe dementia. CNS-related features of the immune system remain hardly understood, and supposed to be “janus-faced”, that is, providing protection as well as causing damage, steered by complex mostly unknown control mechanisms [60, 61]. Protection could be impaired in AD, fueled by—and fueling—debates on possible prevention or therapeutic benefits of antioxidants, radical scavengers, nonsteroidal anti-inflammatory drugs (NSAID), and so forth, although clinical trials do not substantiate the claims.

Microglia are immunocompetent cells that enter brain during developing as immature macrophages. They react rapidly to brain damage, including amyloid pathology in AD. Amyloid deposits attract microglia in a phagosomal attempt of their elimination, but activated microglia release proinflammatory cytokines, chemokines, reactive oxygen species, prostaglandins, and other mediators that harm neurons. Much attention has been paid to neurotoxicity mediated by free radicals, both reactive oxygen and nitrogen species, released by microglia. 

Astrocytes are the most common cell-type in the brain, involved in many functions and increasingly appreciated also in synaptic signaling and integrity. Astrocytes co-localize with microglia at damage-sites, but remain to be explored in detail in AD. Strictly speaking, neurons must also be considered when discussing neuroinflammation because they (can) produce active factors, that is, complement, interleukins, tumor necrosis factor, acute-phase proteins. 

We observed and discussed that degenerating neurons were temporally and spatially closely associated with activated microglia in two independent models, that is, the AAV-Tau viral model and the p25 transgenic model [30, 39]. 

Too many “unknowns” prevent us from answering the question how Tau-mediated damage can lead to neurodegeneration by involving activation of microglia. We maintain the most straightforward hypothesis, that is, that the expression of protein Tau, either wild-type or mutant by the viral vectors, is imposing stress on the pyramidal neurons that cannot adapt rapidly enough to produce Tau-aggregates as a means of temporarily detoxification. The impact on various neuronal functions is evident, including disturbances in post-mitotic state and cell-cycle control, as well as disturbing microtubule-mediated transport. The latter is most destructive in axons and larger dendrites, compromising the major synapses that become dys- or nonfunctional. Secreted neuronal proteins then activate nearby microglia that counter-react by secreting proinflammatory and potentially neurotoxic factors, fueling the viscous cycle that eventually leads to neuronal death. 

## 4. Conclusions

Based on the combination of transgenic and viral models, and taking into account pathological and clinical data from human patients, we defend the thesis that neurodegeneration is not caused by a single defect but by—at least—dual actions (Figure 4). 

The first is proposed to be intrinsically neuronal, for example, damage by accumulation of endogenous proteins in various molecular forms. In the case discussed here, protein Tau is the cause in primary Tauopathies. In AD, the upstream triggers are the amyloid peptides that accumulate because of mutations or other imposed defects in the proteolytic machinery that controls their formation and turnover.

The second factor is also essential, and can be neuronal or microglial in origin, related to any type of stress the brain can experience or has to endure, that is, partial hypoxia, glucose overload or shortage, problems with lipid or cholesterol, or other essential metabolites. The thesis is supported by clinical, but mainly experimental observations. For one, all available data convinced us that the “macro” protein-aggregates, that is, plaques and tangles, are not the most essential in causing cognitive defects and dementia. The challenge to define the neurotoxic Tau-species and its mode of action does nevertheless become not less complicated. 

The combination of transgenic and viral models illustrates eloquently the power of the approach as well as its weaknesses. We still have more modeling to do, taking into account the data and indications that are provided by ongoing clinical studies, because only the human patient can eventually confirm any hypothesis based on experimental models.
